# Interaction between Cannabinoid Compounds and Capsazepine in Protection against Acute Pentylenetetrazole-induced Seizure in Mice

**Published:** 2015

**Authors:** Nima Naderi, Effat Shafieirad, Delaram Lakpoor, Atena Rahimi, Zahra Mousavi

**Affiliations:** a *Department of Pharmacology and Toxicology, School of Pharmacy, Shahid Beheshti University of Medical Sciences, Tehran, Iran.*; b *Neurobiology Research Center, Shahid Beheshti University of Medical Sciences, Tehran, Iran.*; c *Pharmacology and Toxicology Department, Pharmaceutical Sciences Branch and Pharmaceutical Sciences Research Center, Islamic Azad University, Tehran, Iran.*

**Keywords:** Cannabinoid, Capsazepine, Pentylenetetrazole, Seizure, Mice

## Abstract

The pharmacological interaction between cannabinoidergic system and vanilloid type 1 (TRPV1) channels has been investigated in various conditions such as pain and anxiety. In some brain structure including hippocampus, CB1 and TRPV1 receptors coexist and their activation produces opposite effect on excitability of neurons. In this study, we tested the hypothesis that TRPV1 channel is involved in the modulation of cannabinoid effects on pentylenetetrazole (PTZ)-induced seizure threshold. In single therapy, male mice (n = 10 per group) received either TRPV1 receptor antagonist capsazepine, CB1 receptor agonist ACEA or anandamide reuptake inhibitor VDM11. In combination therapy, mice were treated with either capsazepine-ACEA or capsazepine-VDM11 combination prior to seizure test. Thirty min later, mice were submitted to infusion of PTZ (1%, 0.25 mL/min) into tail vein and the dose of PTZ to induce clonic convulsion was considered as seizure threshold. Administration of capsazepine and ACEA per se produced protective effects against PTZ-induced seizure, while administration of VDM11 per se did not produce such a protection effect. The anticonvulsant actions of both capsazepine and ACEA were attenuated after co-administration of these compounds. Moreover, the anticonvulsant action of capsazepine was attenuated after co-administration with VDM11. The results suggest an interaction between cannabinoidergic system and TRPV1 receptors in protection against acute PTZ-induced seizure in mice.

## Introduction

TRPV1 and CB1 receptors share anandamide (AEA) as their common endogenous ligand which indicates a functional interaction between vanilloid and cannabinoid systems(1,2). These two receptors are co-localized in the same cells in many brain structures including cerebral cortex, hippocampus and amygdale (3,4) which are the brain regions involved in seizures. TRPV1 receptors are involved in synaptic plasticity and could potentially contribute to pathological excitation of hippocampal neurons observed in temporal lobe epilepsy (5). On the other hand, several endogenous fatty acids related to cannabinoid system have been identified as TRPV1 agonists (6). The sharing properties of TRPV1 and CB1 receptors in seizure and their common endogenous ligand give reason to suggest a functional interaction between the vanilloid and the cannabinoid systems (1,7). In this study, we showed such an interaction between cannabinoidergic and vanilloidergic systems in pentylenetetrazole-induced acute model of seizure.

## Experimental

*Chemicals*

Following drugs were used in this study: pentylenetetrazole (PTZ), the selective and potent CB1 cannabinoid receptor agonist arachidonyl-2-chloroethylamide (ACEA), the endocannabinoid membrane transporter inhibitor N-(4-hydroxy-2-methylphenyl) arachidonoyl amide (VDM11) and the TPRV1 receptor antagonist capsazepine (CPZ). All drugs were obtained from Sigma Company (St. Louis, MO, USA). ACEA, VDM11, CPZ were initially dissolved in DMSO (purchased from Sigma Company, St. Louis, MO, USA) and final concentrations were obtained by diluting in a vehicle consists of DMSO (33.3%) and normal saline (66.6%). PTZ was dissolved in deionized water. Drugs (except for PTZ) were administered intraperitoneally (*i.p*.) in a volume of 10 mL/Kg.

*Subjects*

Two hundred male NMRI mice weighting 20-30 g (Pasteur Institute, Tehran, Iran) were used in this study. Mice were kept in groups of 10 in plastic cages at controlled temperature (22±2 C) with 12:12h light-dark cycle (lights on at 7:00 a.m.). Animals were allowed free access to food and water. All experiments were conducted between 0900h and 1400h. The experiment group consisted of 10 animals. Each mouse was used only once. Procedures involving animals and their care were conducted in accordance with the Shahid Beheshti Guidelines for Care and Use of Laboratory Animals. All efforts were made to minimize animal suffering and to use only the number of animals necessary to produce reliable scientific data.

*Study design*

In order to investigate the interaction between CPZ and ACEA, each group received two intraperitoneal (*i.p*.) injections in 5 min interval as follows: (i) Control group received CPZ and ACEA vehicles. (ii) Two groups received various doses of CPZ (1 or 10 mg/Kg) along with ACEA vehicle. (iii) Four groups received CPZ vehicle along with various doses of ACEA (1, 2, 4, or 10 mg/Kg). (iv) Four groups received co-administration of CPZ (1 or 10 mg/Kg) and ACEA (1 or 10 mg/Kg). 

In order to investigate the interaction between CPZ and VDM11, mice were randomly selected in nine different groups and each group received two intraperitoneal (*i.p*.) injections in 5 min interval as follows: (i) Control group received CPZ and VDM11 vehicles. (ii) Two groups received various doses of CPZ (1 or 10 mg/Kg) along with VDM11 vehicle. (iii) Two groups received CPZ vehicle along with various doses of ACEA (1 or 10 mg/Kg). (iv) Four groups received co-administration of CPZ (1 or 10 mg/Kg) and VDM11 (1 or 10 mg/Kg).

*PTZ-induced seizure model*

PTZ test was performed 30 min after the last drug injection. The threshold for clonic PTZ-induced seizures was determined by infusion of a 1% solution of PTZ into the tail vein of unrestricted freely moving mice at a constant rate of 0.25 mL/min with an infusion pump (model 53140, Stoelting, USA). The dose of administered PTZ (mg/Kg of mice weight) which produced generalized clonic seizure with loss of righting reflex was considered as the threshold for the clonic seizure.

*Statistical analysis*

Data are expressed as mean± S.E.M. of seizure threshold in each experimental group. Statistical analysis was performed using SPSS16 (SPSS Inc., 2007). The two-way ANOVA followed by Dunnette post test was used to compare the treatment group with control group. *P*<0.05 was considered as statistically significant.

## Results

*Effect of ACEA and CPZ on PTZ-induced seizure threshold*

Administration of various doses of both ACEA and CPZ produced an increase in PTZ-induced seizure threshold in mice. According to the two-way ANOVA analysis, both CPZ [*F*(2,97)=3.518; *p*=0.033] and ACEA [*F*(4,97)=4.517; *p*=0.002] significantly increased PTZ-induced seizure threshold compared to control group. Further analysis by Dunnett’s test showed that ACEA (10 mg/Kg; *p*=0.049) and CPZ (10 mg/Kg; *p*<0.001) significantly increased the required dose of PTZ to induce seizure compared to control group. Furthermore, two-way ANOVA revealed a significant interaction [*F*(4,97)=5.086; *p*<0.001] between ACEA and CPZ in protection against PTZ-induced seizure. Co-administration of ACEA (1 or 10 mg/Kg) and CPZ (1 or 10 mg/Kg) did not produce significant change in PTZ-induced seizure threshold compared to control group (Table 1).

**Table 1:** Effects of various doses of ACEA and capsazepine alone or in combination on PTZ-induced seizure threshold. ACEA or its vehicle was administered 5 min after injection of capsazepine or its vehicle. PTZ-induced seizure test was performed 30 min after administration of ACEA or its vehicle. The control group received capsazepine vehicle and ACEA vehicle. Data are shown as mean ± SEM (N=10).

**ACEA** **(mg/Kg)**	**Capsazepine** **(mg/Kg)**	**PTZ-induced seizure threshold** **(mg/Kg)**
0	0	37.80 ± 2.08
0	1	47.65 ± 1.91
0	10	62.15 ± 4.53 ***
1	0	38.93 ± 4.01
2	0	39.79 ± 2.12
4	0	45.10 ± 2.15
10	0	51.97 ± 2.79 *
1	1	35.52 ± 2.31
10	1	44.75 ± 5.63
1	10	40.03 ± 4.36
10	10	45.95 ± 4.10

* p=0.049 compared to control group

*** p<0.001 compared to control group

*Effect of VDM11 and CPZ on PTZ-induced seizure threshold*

A significant increase in PTZ-induced seizure threshold was observed in mice treated with CPZ compared to control group [*F*(2,81)=9.25; *p*<0.001]. Further analysis by Dunnett’s test revealed a significant effect of CPZ at the doses of 1 mg/Kg (*p*=0.044) and 10 mg/Kg (*p*<0.001). However, administration of VDM11 at the dose of 1 mg/Kg (*p*=0.912) and 10 mg/Kg (*p*=0.959) did not produce significant change in PTZ-induced seizure threshold compared to control group. Moreover, two-way ANOVA revealed a significant interaction between anticonvulsant effects of CPZ and VDM11 in protection against PTZ-induced seizure [*F*(4,81)=4.91; *p*=0.001]. Co-administration of VDM11 (either 1 or 10 mg/Kg) and CPZ (either 1 or 10 mg/Kg) did not produced significant change in PTZ-induced seizure threshold compared to control group (Table 2).

**Table 2:** Effect of various doses of VDM11 and capsazepine alone or in combination on PTZ-induced seizure threshold. VDM11 or its vehicle was administered 5 min after injection of capsazepine or its vehicle. PTZ-induced seizure test was performed 30 min after administration of VDM11 or its vehicle. The control group received capsazepine vehicle and VDM11 vehicle. Data are shown as mean ± SEM (N=10).

**VDM11** **(mg/Kg)**	**Capsazepine** **(mg/Kg)**	**PTZ-induced seizure threshold** **(mg/Kg)**
0	0	35.25 ± 3.30
0	1	47.65 ± 1.91
0	10	62.15 ± 4.53 ***
1	0	31.03 ± 2.27
10	0	38.87 ± 3.79
1	10	35.58 ± 2.65
10	1	38.06 ± 4.29
1	1	28.22 ± 3.61
10	10	37.85 ± 2.37

*** *p* < 0.001 compared to control group.

## Discussion

In the present study, administration of exogenous CB1 receptor agonist ACEA and TRPV1 receptor antagonist CPZ produced protective effect against acute PTZ-induced seizure. However, administration of the cannabinoid reuptake inhibitor VDM11 at the doses of 1 and 10 mg/Kg did not have any effect on this model of seizure. Moreover, co-administration of CPZ with either ACEA or VDM11 antagonized the antiseizure effect of both ACEA and CPZ.

The convulsive action of PTZ is mainly due to its stimulating effect on neurons which is largely mediated through its GABA_A_ receptor antagonist properties (8), although its action on sodium and calcium channels could also give rise to an overall increase in excitability of neurons. Moreover, PTZ can interfere with calcium-dependent cytoplasmic reactions via inhibition of related protein kinase activity (9,10).

The role of TRPV1 receptors in seizure and epilepsy has been shown in many studies [for review, please see 11]. Activation of TRPV1 receptors caused calcium influx into neuron and subsequent increase in calcium dependent kinase activation (12) which favor increase in glutamatergic transmission, post synaptic stimulation (-) and hippocampal network excitability (16). Therefore, TRPV1 channel antagonists could be considered as potential protective drugs (17,11,18), although there is evidence regarding the anti-seizure effects of TRPV1 agonists (19) in both PTZ- and MES-induced seizure models. In our study, pretreatment of mice with TRPV1 receptor antagonist produced protection against PTZ-induced seizure. This action is likely mediated through effects on both transmitter release and cation channels. Although electrophysiological studies assumed that the TRPV1 receptors may presynaptically enhance the release of GABA (20), however, some studies indicate that the TRPV1 receptors play an important role in cellular mechanism underlying a form of LTD triggered at hippocampal glutamatergic synapses on GABAergic interneurons which is thought to underlie synaptic plasticity leads to epilepsy induction (16,21). This type of LTD was blocked by a TRPV1 channel antagonist and was absent in hippocampal slice from TRPV1 knock-out mice (16). 

The concomitant activation of TRPV1 and CB1 receptors leads to either a stronger stimulation of TRPV1 activity or to its inhibition depending on which signaling pathway is activated (22). There are some instances regarding interaction between CB1 and TRPV1 receptors in anxiety-like behavior of experimental animals. Pretreatment of mice with CPZ showed that the anxiolytic effect evoked by AEA might be due to the interaction with the CB1 cannabinoid receptor, whereas TRPV1 receptors seem to be involved in AEA anxiogenic action (23). In other study, it was shown that simultaneous indirect activation of CB1 receptors by FAAH inhibition, and antagonism at TRPV1 receptors produced more anxiolytic action in mice (24). However, in our study, co-administration of CPZ and ACEA did not increase the protective effect of ACEA, but rather attenuated its anticonvulsant action compared to that when administered alone. It could be suggested that while an anticonvulsant action of the exogenous cannabinoid receptor agonist was observed in this study, however, its inhibiting effect on synaptic GABA transmission could interact with the effects of TRPV1 receptor antagonist on PTZ-induced seizure threshold.

Activation of CB1 receptor agonist causes inhibition of both glutamatergic and GABAergic synaptic transmission (25). Prevention of endocannabinoid reuptake by VDM11 increases AEA levels and augments its effect on both CB1 and TRPV1 receptors. Previous studies showed that AEA can activate CB1 receptors at relatively lower doses compared with the dose of AEA required to activate TRPV1 receptor (23). TRPV1channel activation is associated with the increase in excitatory synaptic input to dentate gyrus granule cells in an epilepsy model (26). In this study, augmentation of endocannabinoid levels at synapses induced neither increase nor decrease in PTZ-induced seizure threshold, which could suggest no protective effect of VDM11 against PTZ-induced seizure at least in doses applied in this study. On the other hand, co-administration of VDM11 and CPZ attenuated the anti-seizure action of CPZ. This is likely due to VDM11-induced increased levels of AEA which could surmount CPZ in occupying TRPV1 receptor and eventually attenuate the anti-seizure activity of CPZ.

In conclusion, the results of the present study suggested the protective effect of both exogenous cannabinoid CB1 receptor agonists and TRPV1 channel antagonists in acute PTZ-induced seizure model. However, augmentation of endocannabinoid effect could not achieve a protection against PTZ-induced seizure. Moreover, co-administration of cannabinoids and CPZ attenuated the protective effect of these compounds which could suggest an interaction between the cannabinoid and TRPV1 receptorial systems in pharmacological mechanism of acute PTZ-induced seizure. 
